# PIST regulates the intracellular trafficking and plasma membrane expression of Cadherin 23

**DOI:** 10.1186/1471-2121-11-80

**Published:** 2010-10-19

**Authors:** Zhigang Xu, Kazuo Oshima, Stefan Heller

**Affiliations:** 1 Departments of Otolaryngology - Head & Neck Surgery and Molecular & Cellular Physiology, Stanford University School of Medicine, Stanford, CA 94305, USA; 2 Key Laboratory for Experimental Teratology of the Ministry of Education, Institute of Developmental Biology, School of Life Sciences, Shandong University, Jinan, Shandong 250100, China

## Abstract

**Background:**

The atypical cadherin protein cadherin 23 (CDH23) is crucial for proper function of retinal photoreceptors and inner ear hair cells. As we obtain more and more information about the specific roles of cadherin 23 in photoreceptors and hair cells, the regulatory mechanisms responsible for the transport of this protein to the plasma membrane are largely unknown.

**Results:**

PIST, a Golgi-associated, PDZ domain-containing protein, interacted with cadherin 23 via the PDZ domain of PIST and the C-terminal PDZ domain-binding interface (PBI) of cadherin 23. By binding to cadherin 23, PIST retained cadherin 23 in the trans-Golgi network of cultured cells. The retention was released when either of the two known cadherin 23-binding proteins MAGI-1 and harmonin was co-expressed. Similar to MAGI-1 and harmonin, PIST was detected in mouse inner ear sensory hair cells.

**Conclusions:**

PIST binds cadherin 23 via its PDZ domain and retains cadherin 23 in trans-Golgi network. MAGI-1 and harmonin can compete with PIST for binding cadherin 23 and release cadherin 23 from PIST's retention. Our finding suggests that PIST, MAGI-1 and harmonin collaborate in intracellular trafficking of cadherin 23 and regulate the plasma membrane expression of cadherin 23.

## Background

Cadherins are calcium-dependent transmembrane proteins. They play important roles in cell adhesion, which is crucial for establishing and maintaining tissue architecture and function [1]. Around 80 cadherin proteins have been identified, which can be divided into different subgroups, including classic cadherins, desmogleins, desmocollins, protocadherins, CNRs, Fats, seven-pass transmembrane cadherins, and Ret tyrosine kinase [2]. All cadherins have extracellular cadherin (EC) repeats, the extracellular Ca^2+^-binding domains that mediate cell-cell adhesion, but their cytoplasmic domains are diverse. Classic cadherins (E- and N-cadherins) have a β-catenin-binding motif in their cytoplasmic domain, which is important for the cell adhesion function [2].

The atypical cadherin protein cadherin 23 (CDH23) is closely related to the Fat subgroup, which is characterized by a large number of EC repeats (27 for cadherin 23 and Dachsous, 34 for Fat), a transmembrane domain, and a short cytoplasmic domain. Although it bears no homology with classical cadherins except for the EC repeats, cadherin 23 can mediate cell-cell adhesion when over-expressed in L cells [3]. The cytoplasmic domain of cadherin 23 lacks the β-catenin-binding motif, suggesting that it may not be able to interact with β-catenin directly. However, recently cadherin 23 was shown to bind to the PDZ4 domain of a scaffolding protein, MAGI-1 [4], which in turn can bind to β-catenin via its PDZ5 domain [5], suggesting that MAGI-1 may act as a bridge between cadherin 23 and β-catenin.

The *CDH23 *gene gives rise to different transcripts through two mechanisms. The first mechanism utilizes different promoters, giving rise to proteins with different numbers of EC repeats [6]. The second type involves the alternative splicing of exon 68, which encodes part of the cytoplasmic domain of the cadherin 23(+68) isoform that is preferentially expressed in the inner ear [3,4]. It has been shown that the longest cadherin 23 variant with 27 EC repeats is a part of the tip-links in hair cell stereocilia [3,7], which are the mechanical links that are essential for gating of the mechanoelectrical transduction channels. Mutations of *CDH23 *gene have been identified to associate with blindness and hearing loss [8,9].

Several cadherin 23-binding proteins have been reported, including harmonin [10,11], myosin 1c [3], and protocadherin 15 [7], all of which have been shown to be involved in hearing transduction and/or retinal function. As we learn more and more about the function of cadherin 23, our knowledge about its genesis is limited. Understanding how cadherin 23 is being shuttled to the apical hair cells membrane for example, and ultimately, how tip links are being assembled is crucial for shedding light on the molecular mechanisms of hair cell mechanosensation. Recently, EHD4, a EH domain-containing protein involved in endocytic recycling was identified as a novel cadherin 23-binding partner, and was suggested to play a role in regulating the membrane localization of cadherin 23 [12]. Nevertheless, the regulatory mechanism responsible for the transport of cadherin 23 to the plasma membrane remains unclear.

We have conducted yeast two-hybrid screens of a cochlear cDNA library using the cadherin 23(+68) intracellular domain as a bait, and identified MAGI-1, a MAGUK protein containing multiple PDZ domains, as a novel cadherin 23-interaction partner [4]. Here we report another PDZ domain-containing protein identified from the screen, PIST (also known as GOPC, or CAL). PIST was first reported as a putative binding protein of the Rho protein TC10, which is reflected in its name PIST: PDZ domain protein interacting specifically with TC10 [13]. As a Golgi-associated protein, PIST has been shown to interact with some transmembrane proteins and regulate the intracellular sorting and plasma membrane expression of these proteins [14-19].

Here we showed that PIST and cadherin 23 interact via the PDZ domain of PIST and the cytoplasmic PDZ-binding interface (PBI) of cadherin 23, respectively. When co-expressed in cultured cells, PIST retained cadherin 23 in trans-golgi networks (TGN). MAGI-1 and harmonin, two known cadherin 23-binding proteins that are expressed in hair cells were able to compete with PIST and to release cadherin 23 from its retention. Immunostaining showed that PIST is expressed in inner ear hair cells. Our results suggest that PIST, MAGI-1 and harmonin regulate the intracellular sorting of cadherin 23, thereby affecting the membrane localization of cadherin 23.

## Methods

### Yeast Two-hybrid Screen

The yeast two-hybrid screen was performed as described before [4]. Briefly, a chicken basilar papilla cDNA library [20] was screened using the carboxyl-terminal 265 amino acids (aa) of chicken cadherin 23(+68) as a bait. 5 × 10^6 ^total transformants were selectively screened using *HIS3 *(at the presence of 2.5 mM of 3-amino-1,2,4-triazole) as the primary reporter gene, then two more reporter genes *ADE2 *and *lacZ *were used to verify the positive colonies. The prey vectors in triple-positive yeast colonies were recovered and cDNA inserts were sequenced.

### Expression Vectors

Mouse CDH23 cDNA is a gift from K. Noben-Trauth (National Institute on Deafness and Other Communication Disorders, Rockville, MD), which consists of CDH23 cDNA encoding the first three extracellular cadherin (EC) repeats (1-348 aa) fused to cDNA encoding the protein's carboxyl-terminus (2975-3354aa in cadherin 23(+68), 2975-3319aa in cadherin 23(-68)) including the transmembrane domain. For protein manipulation, we added His and c-Myc tags between the signal peptide and the first EC repeat. The cDNA encoding cadherin 23 lacking the last 4 aa (ITEL) at the carboxyl-terminus was PCR amplified and cloned into pcDNA3.1(+) to generate expression vectors for Myc-cadherin 23 (-ITEL). Human HA-PIST cDNA is a gift from Dr. W. B. Guggino (Johns Hopkins Medical Institute, Baltimore, MD). PIST cDNA was PCR amplified and cloned into pEGFP-C2 to express full length PIST, PIST CC2-plus domain (146-274aa), and PIST PDZ domain (276-366aa) as EGFP fusion proteins. Mouse MAGI-1c cDNA is a gift from K. M. Patrie (University of Michigan, Ann Arbor, MI), and was PCR-amplified and cloned into pEGFP-C2 for the expression of EGFP-MAGI-1c protein. Mouse harmonin cDNA was PCR-amplified from mouse organ of Corti cDNA and cloned into pEGFP-C2 for the expression of EGFP-harmonin protein.

### Co-immunoprecipitation

HEK293 cells were transfected with the expression vectors using GeneJammer transfection reagent (Stratagene, La Jolla, CA). Transfected cells were washed with PBS 24-48 hours after transfection and lysed in ice-cold lysis buffer consisting of 150 mM NaCl, 50 mM Tris at pH 7.5, 1% (vol/vol) Triton X-100, 1 mM PMSF, and 1 × protease inhibitor cocktail (Sigma-Aldrich, Saint Louis, MO). For immunoprecipitation, we used immobilized monoclonal anti-c-Myc agarose beads (Sigma-Aldrich) and performed the experiments according to the manufacturer's recommendation. Following 2 hours of incubation at 4°C, immunoprecipitated proteins were washed five times with washing buffer (a modified lysis buffer containing 500 mM NaCl instead of 150 mM), separated by polyacrylamide gel electrophoresis, then transferred to nitrocellulose membrane. The proteins were probed with corresponding antibodies and detected with an Odyssey Infrared Imaging System (LI-COR Biosciences, Lincoln, NE).

### Immunocytochemistry

All steps were performed at room temperature unless otherwise indicated. Transfected cells (HEK293, CHO, or COS-7) growing on Gelatin-coated glass cover slips were fixed with 4% paraformaldehyde (PFA) in phosphate-buffered saline (PBS) for 15 minutes, then permeabilized and blocked with PBT1 (0.1% Triton X-100, 1% BSA, 5% heat-inactivated goat serum in PBS, pH 7.3) for 30 minutes, followed by incubation with mouse anti-myc antibody 9E10 (The Developmental Studies Hybridoma Bank, Iowa City, IA) and/or rabbit anti-PIST antibody (Affinity purified, a gift from Dr. K. Nagata (Institute for Developmental Research, Aichi Human Service Center, Kasugai, Japan)), 1:500 diluted in PBT1, over night at 4°C. In other experiments, polyclonal rabbit antibody against cadherin 23 (a gift from Dr. Ulrich Muller (Scripps Research Institute, La Jolla, CA)) and monoclonal mouse antibody against golgin-97 (A-21270, Invitrogen, Carlsbad, CA), 1:200 diluted in PBT1, were used to label cadherin 23 and trans-golgi network marker golgin-97. After washing twice with PBT1 for 10 minutes and twice with PBT2 (0.1% Triton X-100, 0.1% BSA in PBS) for 5 minutes, cells were incubated with 7.5 μg/ml fluorescence-conjugated secondary antibody (Jackson ImmunoResearch Inc., West Grove, PA) in PBT2 for 1 hour, followed by two 5 minutes PBT2 washes and two 5 minutes PBS washes. For nuclei staining, cells were then incubated with TOTO-3 (Molecular Probes, Eugene, OR), 1:2,000 diluted in PBS for 1 hour, followed by three 10 minutes PBS washes, then mounted in Glycerol/PBS (1:1). Immunostaining was imaged with a confocal microscope (LSM Pascal, Zeiss, Germany).

### Tissue section staining

All animal procedures followed guidelines set forth by the National Institutes of Health. The cochleae were dissected from P5 and adult (4-week old) C57 mice, immediately fixed in 4% PFA for 3 hours, then immersed in 30% sucrose solution overnight and embedded in O.C.T. compound (Tissue-Tek, Sakura Finetek, Japan). Blocks were then frozen at -20°C and sectioned at 14 μm thickness onto slide glasses with Cryostat (Leica CM3050S, Germany). Staining was performed as described above. Briefly, sectioned tissues were incubated with rabbit anti-PIST antibody (1:100) and guinea pig anti-myosin VIIa antibody (1:200) [21], then 7.5 μg/ml fluorescence-conjugated secondary antibody (Jackson ImmunoResearch Inc.). After the final wash with PBS, tissues were stained with TRITC-conjugated phalloidin (Sigma-Aldrich) to visualize F-actin in the hair bundles of hair cells and DAPI (Molecular Probes) to visualize nuclei. The slides were analyzed by fluorescence microscopy and digital image acquisition (Zeiss Axioimager and AxioCam).

## Results

### PIST's interaction with cadherin 23 is PDZ mediated

**Figure 1:**
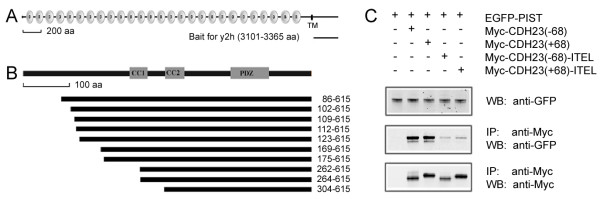
**Interaction between PIST and cadherin 23**. A. Schematic diagram of chicken cadherin 23 domain structure and the intracellular fragment used as the bait for yeast two-hybrid screens. B. Schematic diagram of chicken PIST domain structure and PIST protein fragments encoded by the clones isolated from yeast two-hybrid screens. C. Western blots showing co-immunoprecipitation (co-IP) of EGFP-tagged human PIST protein with Myc-tagged mouse cadherin 23(+68) and cadherin 23(-68). The co-IP is almost undetectable when the last 4 amino acids (ITEL) of cadherin 23 are deleted. IP indicates antibody used for immunoprecipitation and WB indicates antibody used for detection.

To identify proteins that interact with cadherin 23, we performed yeast two-hybrid screens of a chicken basilar papilla cDNA library using the intracellular part of chicken cadherin 23(+68) protein as a bait (Figure 1A). Proteins identified through the yeast two-hybrid screen include scaffolding proteins (for example, MAGI-1 [4]) and chaperones (such as PIST, see below), most but not all of which are PDZ domain-containing proteins. Twenty-nine clones, which account for more than half of all the isolated positive clones, encode the chicken PDZ-containing, Golgi-associated chaperone protein PIST. The isolated 29 PIST clones fall into 10 groups of unique cDNAs, covering different lengths of PIST's amino acid sequence (Figure 1B). The longest clone encodes nearly the full-length chicken PIST, only missing the N-terminal 85 amino acids comparing to the predicted chicken PIST sequence in NCBI database, and the shortest contains the second coiled-coil domain and the PDZ domain.

**Figure 2:**
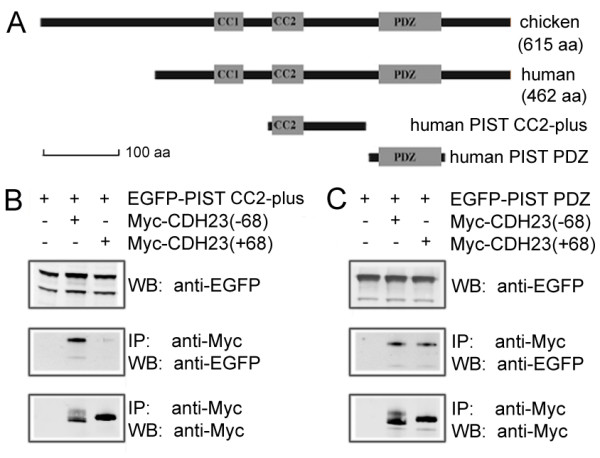
**PIST binds to cadherin 23 via its PDZ domain, as well as the CC2-plus region**. A. Schematic diagram of chicken and human PIST domain structures, and the domains of human PIST used in co-IP experiments. B. EGFP-tagged PIST CC2-plus region is co-IPed with Myc-tagged cadherin 23(-68), but not cadherin 23(+68). C. EGFP-tagged PIST PDZ domain is co-IPed with Myc-tagged cadherin 23(+68) and cadherin 23(-68)

**Figure 3:**
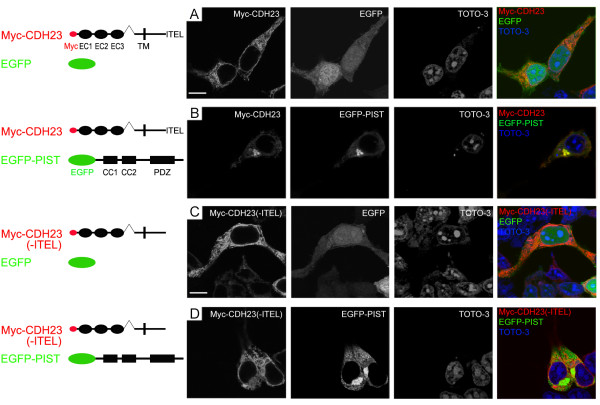
**Co-localization of cadherin 23 with PIST in transfected HEK293 cells**. A. Myc-tagged cadherin 23 localizes in the cytoplasm. B. In the presence of EGFP-tagged PIST, Myc-tagged cadherin 23 co-localizes with EGFP-PIST at TGN-like structures. C. Myc-tagged cadherin 23 without the last 4 amino acids (ITEL) localizes in the cytoplasm. D. In the presence of EGFP-PIST, Myc-tagged cadherin 23 without the last 4 amino acids (ITEL) remains in the cytoplasm, albeit EGFP-PIST localizes at TGN-like structures. Myc-cadherin 23 was stained with mouse anti-Myc antibody and then TRITC-conjugated goat anti-mouse secondary antibody. Nuclei were stained with TOTO-3. Scale bars, 10 μm.

To verify the interaction biochemically, we performed co-immunoprecipitation (co-IP) experiments. Both proteins are highly conserved among different species. Mammalian cadherin 23 and PIST share about 90% homology with their chicken counterparts. Comparing to the predicted chicken PIST protein, mammalian PIST misses about 150 aa at the N-terminus (Figure 2A). There are no predicable domains in this region. Mammalian hair cells are becoming more and more the main focus of research, which prompted us to focus the rest of the investigation on mammalian proteins. The full-length *CDH23 *cDNA is more than 10 kb long and difficult to manipulate, so instead of using the full-length cDNA, we made a construct that expresses a Myc-tagged cadherin 23 protein missing EC4-27 (Figure 3, left panel). When over-expressed in HEK293 cells, EGFP-tagged human PIST was co-immunoprecipitated with Myc-tagged mouse cadherin 23, and this interaction was not affected by the splicing of exon 68 (Figure 1C). Co-IP experiments with EGFP-tagged PIST coiled-coil domain 2 (CC2) plus the amino acids between CC2 and the PDZ domain (CC2-plus, 146-274aa) or the PDZ domain (276-366aa) revealed that both domains were co-IPed with Myc-cadherin 23 (Figure 2B,C). The interaction between PIST's PDZ domain and cadherin 23 was not affected by the splicing of exon 68, while the interaction between PIST CC2-plus and cadherin 23 was isoform-dependent, with the cadherin 23(-68) interaction being much stronger than cadherin 23(+68). The interaction between PIST CC2-plus and cadherin 23(+68) was barely detectable.

The last 4 amino acids (ITEL) at cadherin 23's carboxyl-terminus constitute a class-I PDZ domain binding interface (PBI), which is important for the interaction of cadherin 23 with two other PDZ domain containing proteins, harmonin and MAGI-1 [4,11]. We then tested whether these 4 amino acids are also important for the association of cadherin 23 with PIST. We found that cadherin 23 lacking the last 4 amino acids (cadherin 23(-ITEL)) displayed decreased binding ability to PIST when compared to intact cadherin 23 (Figure 1C). Although the removal of binding could be the consequence of misfolding of the truncated protein, our data as well as other published results suggested that the principal interaction between the two proteins utilizes the PDZ binding interface.

### PIST retains cadherin 23 in the trans-Golgi network

**Figure 4:**
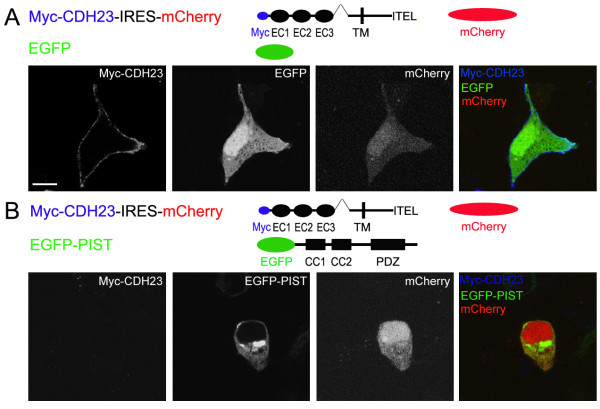
**PIST reduced the membrane expression of cadherin 23 in transfected HEK293 cells**. A. Myc-cadherin 23 immunoreactivity using a non-permeable protocol shows clear plasma membrane localization. B. In the presence of EGFP-PIST, Myc-cadherin 23's plasma membrane immunoreactivity is undetectable. Staining was performed as normal except Triton X-100 was excluded. Myc-cadherin 23 was stained with mouse anti-Myc antibody and then Cy5-conjugated goat anti-mouse secondary antibody. mCherry was used as a control indicating Myc-cadherin 23-IRES-mCherry cassette was expressed successfully. Scale bar, 10 μm.

It has been shown that PIST can regulate the subcellular localization of its interacting partners. To test whether this also applies to cadherin 23, we analyzed the subcellular distribution of PIST and cadherin 23 in transfected HEK293 cells by immunocytochemistry and confocal microscopy. Myc-cadherin 23 was found in the cytoplasm as well as associated with the plasma membrane (Figure 3A). The association with the plasma membrane was clearly visible when we stained for the extracellular Myc epitope of Myc-cadherin 23 without permeabilizing the cells (Figure 4A). The Myc epitope is placed in the extracellular part of cadherin 23, so when a non-permeable protocol is used, only the plasma membrane cadherin 23 can be visualized. When co-expressed with EGFP-PIST, however, Myc-cadherin 23 was found to co-localize with EGFP-PIST (Figure 3B), which exhibited a TGN-like staining pattern, and the plasma membrane expression of cadherin 23 was dramatically reduced to an undetectable level (Figure 3B and 4B). To make sure that the reduced membrane expression of Myc-cadherin 23 in Figure 4B is not caused by unsuccessful transfection, we used a construct that expresses Myc-cadherin 23 and mCherry at the same time (Myc-cadherin 23-IRES-mCherry), so when mCherry is detected in a cell, Myc-cadherin 23 is expressed in the same cell. When we disrupted the interaction between these two proteins by removing the last 4 amino acids of cadherin 23, the subcellular localization of cadherin 23(-ITEL) was not affected by EGFP-PIST any more (Figure 3C and 3D).

**Figure 5:**
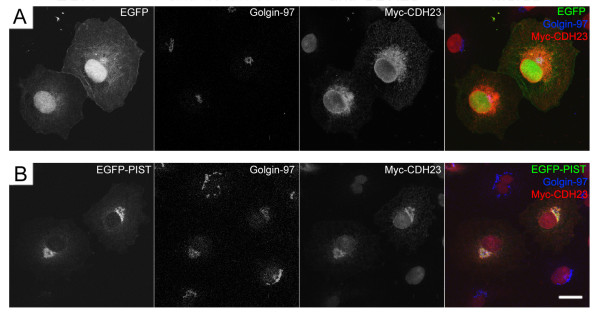
**Retention of cadherin 23 by PIST in trans-golgi network in transfected COS-7 cells**. A. Myc-tagged cadherin 23 localizes in the cytoplasm. B. In the presence of EGFP-tagged PIST, Myc-tagged cadherin 23 co-localizes with EGFP-PIST and a trans-golgi network marker golgin-97. Myc-cadherin 23 was stained with rabbit anti-cadherin 23 antibody and golgin-97 was stained with mouse anti-golgin-97 antibody. Scale bar, 20 μm.

The retention of cadherin 23 by PIST was also observed in transfected CHO and COS-7 cells (data not shown). PIST has been shown to associate with trans-golgi network (TGN) in mammalian cells [14]. To explore whether PIST retains cadherin 23 in TGN, we stained transfected cells with an antibody against a TGN maker, glogin-97. When cotransfected in COS-7 cells, Myc-cadherin 23 co-localized with EGFP-PIST, as well as golgin-97 (Figure 5). Our data suggests that by binding to cadherin 23, PIST retains cadherin 23 in TGN, hence reduces its membrane expression.

### MAGI-1 and harmonin compete with PIST, and release cadherin 23 from trans-Golgi network retention

**Figure 6:**
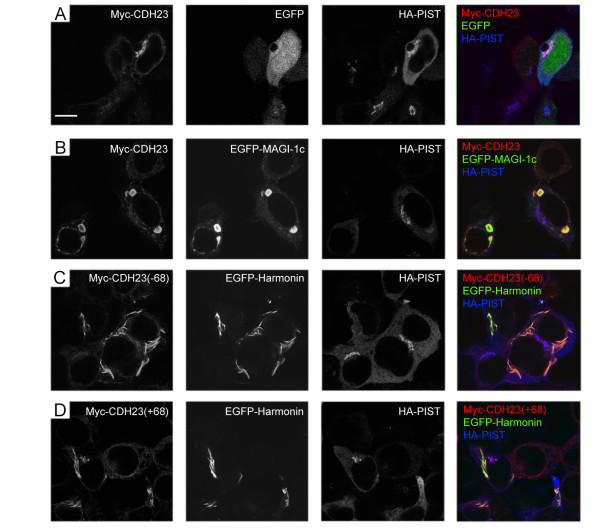
**MAGI-1 and harmonin release cadherin 23 from PIST's retention in transfected HEK293 cells**. A. Myc-tagged cadherin 23 co-localizes with HA-tagged PIST at TGN-like structures. B. In the presence of EGFP-MAGI-1c, Myc-cadherin 23 colocalizes in the cytoplasm with EGFP-MAGI-1c, instead of co-localizing with HA-PIST at TGN-like structures. C. In the presence of EGFP-harmonin, Myc-cadherin 23(-68) co-localizes with EGFP-harmonin, instead of co-localizing with HA-PIST at TGN-like structures. D. In the presence of EGFP-harmonin, Myc-cadherin 23(+68) co-localizes with EGFP-harmonin, as well as with HA-PIST at TGN-like structures. Myc-cadherin 23 was stained with mouse anti-Myc antibody then TRITC-conjugated goat anti-mouse secondary antibody. HA-PIST was stained with rabbit anti-PIST antibody then Cy5-conjugated goat anti-rabbit secondary antibody. Scale bar, 10 μm.

MAGI-1, harmonin, and PIST can independently interact with cadherin 23 via a PDZ domain-mediated mechanism [4,10,11]. Given the fact that they all bind to the same binding site at the carboxyl-terminus of cadherin 23 (harmonin can also bind to an internal peptide of cadherin 23(-68) [22]), a single cadherin 23 protein might only be able to bind to either PIST, or MAGI-1, or harmonin. We consequently explored whether MAGI-1 or harmonin compete with PIST for binding cadherin 23, and furthermore, whether they are able to release cadherin 23 from the PIST-mediated trans-Golgi network retention. EGFP-MAGI-1 exists as protein aggregates in the cytoplasm when overexpressed in HEK293 cells (Figure 6B). When we co-expressed Myc-cadherin 23 and HA-PIST in presence of EGFP-MAGI-1, Myc-cadherin 23 (both +68 and -68 isoforms) co-localized with EGFP-MAGI-1 in the cytoplasm, and was no longer associated with HA-PIST in the trans-golgi network (Figure 6A,B). This suggests that MAGI-1 is able to competitively displace PIST from binding to cadherin 23, and that this competition releases the retention of cadherin 23 in the trans-golgi network.

The interaction between harmonin and cadherin 23 is more complex, involving multiple binding sites. The PDZ2 domain of harmonin binds weakly to cadherin 23's carboxyl-terminus. A second interaction happens between the region immediately upstream of harmonin's PDZ1 domain and an internal peptide of cadherin 23's intracellular domain [22]. The amino acids encoded by exon 68 are adjacent to this binding site in cadherin 23 and may affect the binding to harmonin, since it has been shown that harmonin binds to cadherin 23(-68) much more robustly than to cadherin 23(+68) [4,11]. This preferential splice variant-dependent binding was functionally confirmed in our subcellular localization assays. When Myc-cadherin 23(-68) and HA-PIST were co-expressed in HEK293 cells in presence of EGFP-harmonin, Myc-cadherin 23(-68) co-localized with EGFP-harmonin, which was associated with filamentous structures in the cytoplasm as described before [10], but not in trans-golgi networks where HA-PIST localizes (Figure 6C). Conversely, when Myc-cadherin 23(+68) and HA-PIST were co-expressed in HEK293 cells in presence of EGFP-harmonin, Myc-cadherin 23(+68) displayed a mixed localization, partially colocalized with EGFP-harmonin in the cytoplasm, as well as partially colocalized with HA-PIST in the trans-golgi network, which is consistent with a weaker binding ability of cadherin 23(+68) to harmonin (Figure 6D).

### PIST protein is expressed by all types of hair cells

**Figure 7:**
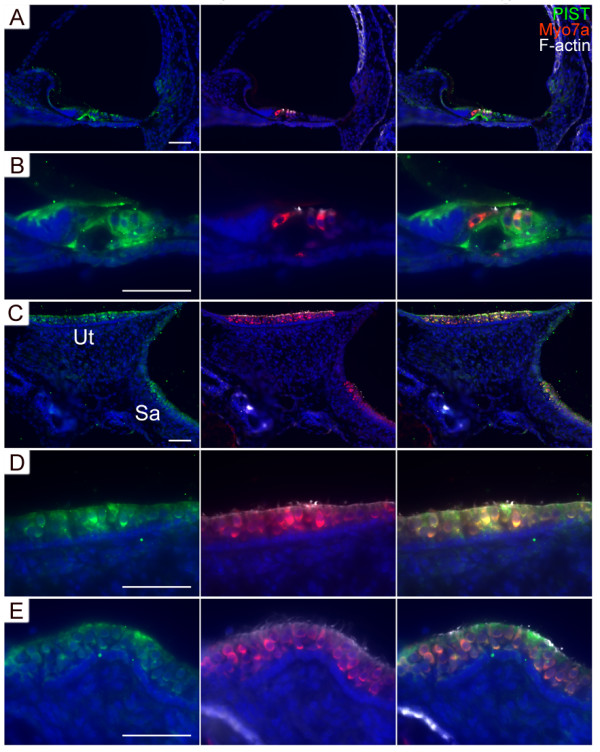
**PIST is expressed in P5 mouse inner ear**. (A) Mouse organ of Corti immunolabeled with rabbit antibodies to PIST (detected with FITC-conjugated secondary antibodies, green) and with guinea pig antibodies to myosin VIIa (Myo7a, detected with Cy5-conjugated secondary antibodies, red). (B) Mouse organ of Corti at higher magnification reveals PIST immunoreactivity in organ of Corti supporting cells, outer hair cells (OHC), and albeit weaker, in inner hair cells (IHC). The extracellular tectorial membrane (TM) shows unspecific labeling with the PIST antibody. (C) Mouse utricle and saccule staining. (D) Mouse utricle staining at higher magnification. (E) Mouse ampulla staining. DAPI (blue) was used to label cell nuclei. TRITC-conjugated Phallodin (white) was used to label stereocilia. Scale bars, 50 μm.

Cadherin 23 expression is restricted to a few cell types; cadherin 23(+68) especially has only been detected in inner ear sensory hair cells so far [3,4]. If PIST functionally interacts with cadherin 23, we would expect it to be expressed in these cell types. We used affinity-purified antibodies to examine the expression pattern of PIST in the mouse inner ear. In the cochlea of postnatal day 5 (P5) mice, PIST immunoreactivity was detectable in inner and outer hair cells as well as supporting cells, including pillar cells and Deiter's cells (Figure 7A,B). Also in the five vestibular organs (utricle, saccule, and the three ampullae of the semicircular canals), PIST immunoreactivity was confined to the sensory epithelia, where it labeled hair cells and probably also supporting cells (Figure 7C-E). In the adult mouse cochlea, PIST shows a similar expression pattern (data not shown).

## Discussion

Our findings support the hypothesis that the Golgi-associated, PDZ domain-containing protein PIST, in conjunction with two other PDZ domain-containing proteins MAGI-1 and harmonin, plays roles in trafficking cadherin 23 to different subcellular locations. It has been shown that PIST interacts with some membrane proteins, and regulates the intracellular trafficking and localization of these membrane proteins: over-expression of PIST leads to a dramatic decrease in the plasma membrane expression of CFTR [15], ClC-3 chloride channels [14], the β1 adrenergic receptor [23], and the somatostatin receptor subtype 5 [18]. Here we show that over-expression of PIST retains cadherin 23 in the trans-golgi network and decreases the plasma membrane expression of cadherin 23, suggesting that it is able to regulate the intracellular localization and/or sorting of cadherin 23. This regulation requires the C-terminal PDZ domain-binding interface (PBI) of cadherin 23.

Interestingly, we found that besides the PDZ domain, the CC2 domain and its downstream amino acids (PIST CC2-plus) also interacts with cadherin 23 in an isoform-dependent way. Nevertheless, this binding site does not appear to mediate the principal interaction between these two proteins because interference of the PDZ/PBI-mediated interaction between PIST and cadherin 23 abolishes the interaction of these two proteins nearly completely.

We also found that the other two cadherin 23-binding, PDZ domain-containing proteins, MAGI-1 and harmonin, can compete with PIST in cellular assays, resulting in release of cadherin 23 from trans-golgi networks. Consistent with its equal binding strength to cadherin 23(+68) and cadherin 23(-68), MAGI-1 can release both cadherin 23 isoforms from PIST's retention. On the other hand, harmonin has a lower binding ability to cadherin 23(+68) when compared to cadherin 23(-68), hence although it releases cadherin 23(-68) efficiently, it only partially releases cadherin 23(+68) from PIST's retention.

We used HEK293 cells as a model to study the competitions among these PDZ domain-containing proteins for binding cadherin 23. In polarized cells such as hair cells, both MAGI-1 and harmonin have been shown to associate with the plasma membrane and hair cell stereocilia [4,10,24]. In our study, over-expressed MAGI-1 and harmonin are not targeted to the membrane; instead, they showed cytoplasmic localization and aggregation. This mislocalization is likely the result of overexpression in a cell line. In addition, other unknown factors that are potentially present in hair cells might contribute to the membrane localization of MAGI-1 and harmonin. Nevertheless, our data provide the first clues on posttranslational targeting of cadherin 23 through the trans-golgi network to other places inside the cell. We hypothesize that PIST in hair cells can transiently interact with cadherin 23 and retain cadherin 23 in the trans-golgi networks, and that this retention is released by either MAGI-1 or harmonin. We speculate that MAGI-1/cadherin 23 or harmonin/cadherin 23 are targeted together to the apical hair cell plasma membrane.

Cadherin 23 has a limited expression profile, only detected in some cell types. Cadherin 23(+68), which is suggested to be the hair cell tip-link component, is only reported in hair cells so far [3]. In mouse inner ear, PIST expression was restricted to hair cells and supporting cells, both in the auditory and vestibular systems. This places all four proteins, cadherin 23, PIST, harmonin, and MAGI-1 into hair cells. It has been shown that in hair cells, cadherin 23 is detected on the stereocilia, and not detectable in the trans-golgi network, but it is very clear the native cadherin 23 needs to pass through the TGN (and very likely interacts with other proteins in the TGN) on its way to the apical hair cell plasma membrane. Our data suggests that PIST may play an important role in regulating the intracellular sorting/localization of cadherin 23. PIST, harmonin, and MAGI-1, the three cadherin 23-binding, PDZ domain-containing proteins, may work together, perhaps sequentially, to regulate cadherin 23's transport to the plasma membrane.

## Conclusions

Our data suggests a possible regulatory mechanism responsible for the transport of cadherin 23 to the plasma membrane. We show that cadherin 23 interacts with PIST, a Golgi-associated, PDZ domain-containing protein, which retains cadherin 23 in the trans-golgi network, and reduces the membrane expression of cadherin 23. In this way PIST plays a negative role in targeting cadherin 23 to the plasma membrane. We also show that MAGI-1 and harmonin can compete with PIST for binding cadherin 23 and release cadherin 23 from PIST's retention. Taken together, PIST, MAGI-1 and harmonin may collaborate in intracellular trafficking of cadherin 23 and regulate the plasma membrane expression of cadherin 23.

## List of abbreviations

CDH23: cadherin 23; EC: extracellular cadherin; PBI: PDZ binding interface; TGN: trans-golgi network; HEK: human embryonic kidney; Co-IP: co-immunoprecipitation; PBS: phosphate buffered saline; PFA: paraformaldehyde; CC2: coiled-coil domain 2; TM: tectorial membrane.

## Authors' contributions

ZX performed the yeast two-hybrid experiment, the co-immunoprecipitation experiment, and the immunochemistry experiment. KO performed the tissue section staining experiment. ZX and SH designed the experiments, analyzed the results and wrote the manuscript. All authors read and approved the final manuscript.
